# *Spirometra* (Pseudophyllidea, Diphyllobothriidae) Severely Infecting Wild-Caught Snakes from Food Markets in Guangzhou and Shenzhen, Guangdong, China: Implications for Public Health

**DOI:** 10.1155/2014/874014

**Published:** 2014-01-16

**Authors:** Fumin Wang, Weiye Li, Liushuai Hua, Shiping Gong, Jiajie Xiao, Fanghui Hou, Yan Ge, Guangda Yang

**Affiliations:** ^1^Guangdong Provincial Wildlife Rescue Center, Guangzhou 510520, China; ^2^Guangdong Entomological Institute (South China Institute of Endangered Animals), No. 105, Xin Gang Road West, Guangzhou 510260, China

## Abstract

Sparganosis is a zoonotic disease caused by the spargana of *Spirometra*, and snake is one of the important intermediate hosts of spargana. In some areas of China, snake is regarded as popular delicious food, and such a food habit potentially increases the prevalence of human sparganosis. To understand the prevalence of *Spirometra* in snakes in food markets, we conducted a study in two representative cities (Guangzhou and Shenzhen), during January–August 2013. A total of 456 snakes of 13 species were examined and 251 individuals of 10 species were infected by *Spirometra*, accounting for 55.0% of the total samples. The worm burden per infected snake ranged from 1 to 213, and the prevalence in the 13 species was 0**∼**96.2%. More than half (58.1%) of the spargana were located in muscular tissue, 25.6% in subcutaneous tissue, and 16.3% in coelomic cavity. The results indicated that *Spirometra* severely infected snakes in food markets in Guangzhou and Shenzhen, implying that eating snakes has great health risk and improper cooking methods may increase the risk of *Spirometra* infection in humans in China. Additional steps should be considered by the governments and public health agencies to prevent the risk of snake-associated *Spirometra* infections in humans.

## 1. Introduction

Spargana of *Spirometra* can parasitize in human body and result in sparganosis, which is an important foodborne parasitic zoonosis [1]. There are three hosts through the life cycle of *Spirometra*, including two intermediary hosts and a definitive host. The first intermediate host is the small crustaceans (*Cyclops* genus); then tadpoles, frogs, fish, and snakes could be infected by *Spirometra* and become the second intermediate hosts; finally, carnivores such as birds, dogs, and cats serve as the definitive hosts of *Spirometra* [2]. Humans are accidental hosts taking the place of either the second intermediary host or of the definitive host, notably by consuming raw meat [3].

Sparganosis has been reported in 39 countries in the world, and it mainly occurs in east and southeast Asia and has also been reported in Europe, America, Africa, and Australia [4]. In China, human sparganosis has been reported in 25 provinces [5]. The first case of human sparganosis was reported in 1882 in Xiamen of Fujian Province, China [6]. To date, over 1000 cases of human sparganosis have been reported in mainland China, of which, 10% of the cases occurred in Guangdong Province [7].

The high prevalence of sparganosis in Guangdong Province may be related to the local dietary habit, where snake is regarded as popular delicious and nutritious food [8, 9]. In Guangzhou, about half of the local restaurants provide wild-caught snakes, and the annual trade volume ever reached 3,612 tons [8, 10]. To make matters worse, many people enjoy eating halfcooked or even completely raw meat/skin/gall bladder of snakes, without considering the high risk of infection by parasites.

Based on the high prevalence of sparganosis and the unhealthy habit of eating snakes in Guangdong, we conducted this survey to further understand the prevalence of *Spirometra* infection in common snakes in food markets. The purpose of this study was to assess the risks of human spargana infection caused by the consumption of wild-caught snakes and provide scientific foundation for preventing the human sparganosis.

## 2. Materials and Methods

### 2.1. Snake Samples

The survey was conducted between January and August of 2013. A total of 456 snake samples (252 living and 204 frozen snakes) were selected from the seized snakes from food markets in Guangzhou and Shenzhen, South China. The snake samples were kept in the Guangdong Provincial Wildlife Rescue Center by local wildlife management department. With the permission from local wildlife management department, we conducted this work. The living snake samples were euthanized using ethyl ether anesthesia before checking spargana. Snake species were identified according to their morphological characteristics [11].

### 2.2. Parasite Inspection

The specimens were dissected to examine the infection by *Spirometra* according to the methods of Wang et al. [12]. Their body length and weight were measured before dissection. For each individual, the skin was entirely peeled off from neck to the tip of tail and the visceral mass from the esophagus and trachea to the cloaca was isolated from the body. Then, the number of *Spirometra* located in muscle tissue, subcutaneous tissue, and coelom (including viscera) was respectively counted in order to investigate the distribution of *Spirometra* inside the snake body. The data was processed with Excel 2007 Software and SPSS. The nonparametric Kruskal-Wallis test was used to compare the difference of the number of worms among the muscle tissue, subcutaneous tissue, and coelom of snakes.

## 3. Results

These selected snake samples composed 13 species, including *Deinagkistrodon acutus*, *Bungarus multicinctus*, *Naja atra*, *Dinodon rufozonatum*, *Elaphe carinata*, *E. taeniura*, *Enhydris bocourti*, *En. chinensis*, *En. plumbea*, *Ptyas korros*, *P. mucosus*, *Xenochrophis piscator*, and *Zoacys dhumnades*. The body length of the 456 snake samples ranged from 27 to 240 cm and the body weight ranged from 15.1 to 2,346.0 g. Essential information including sample source, number of samples of each species, body length and weight of each snake was shown in Table 1.

**Figure 1:**
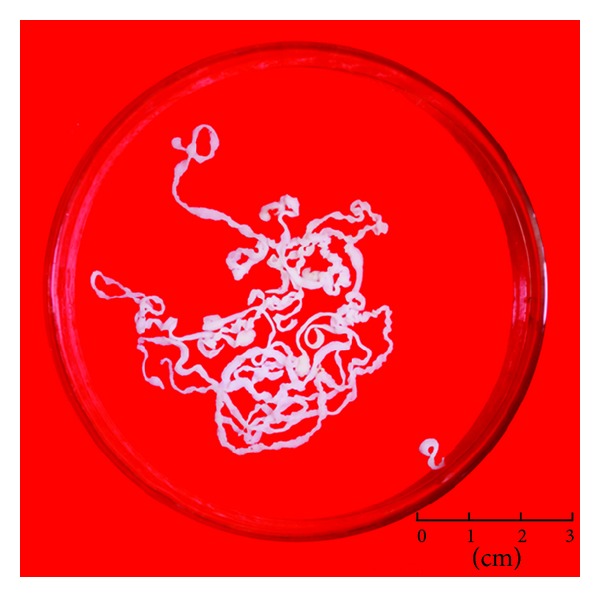
The exterior view of *Spirometra* isolated from snakes.

**Figure 2:**
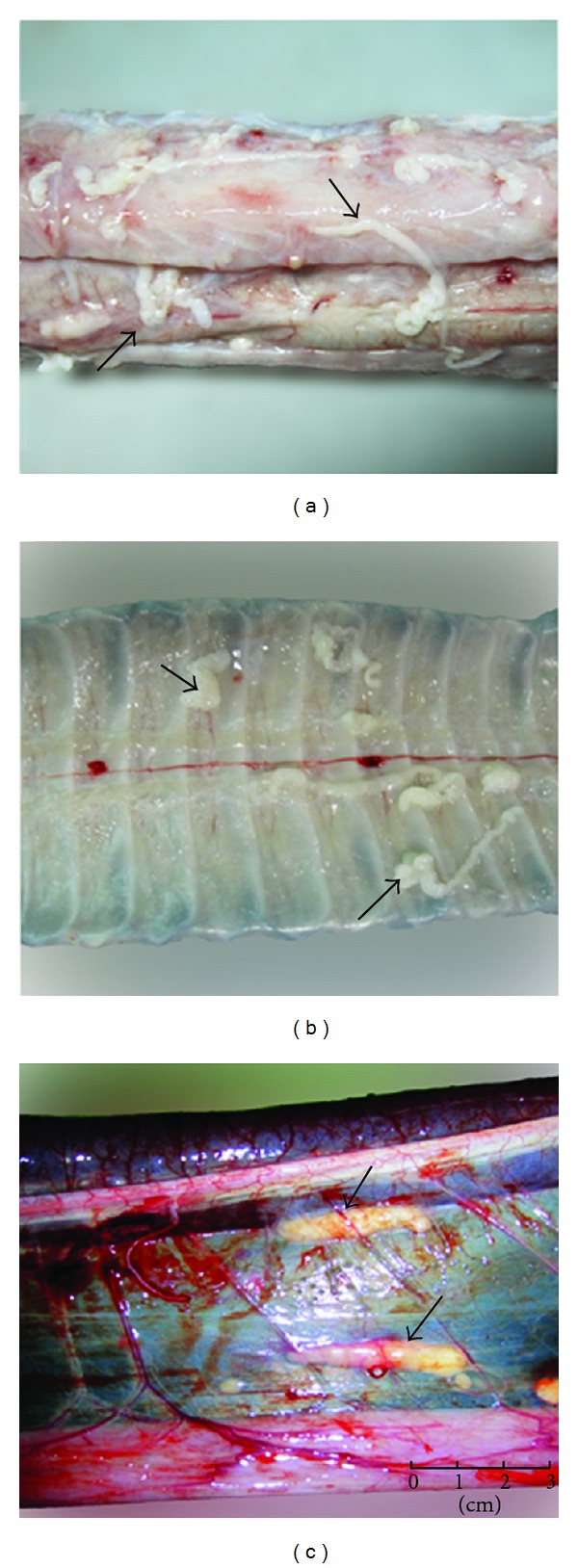
*Spirometra* located in muscle tissue (a), subcutaneous issue (b), and coelom (c) of *Zoacys dhumnades*. Arrows point to *Spirometra*.

**Figure 3:**
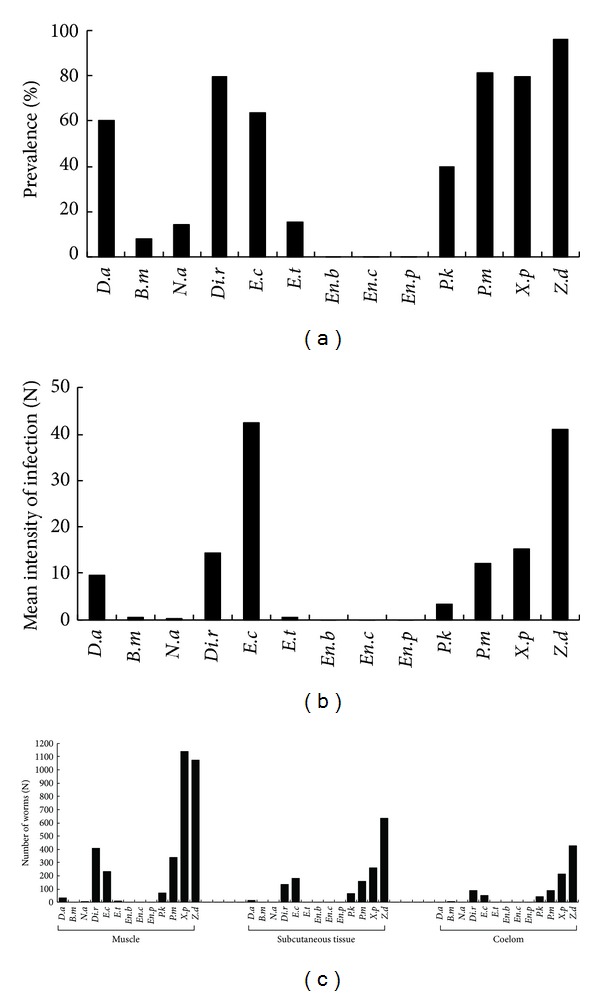
Prevalence (a), intensity (b), and parasitizing locations (c) of *Spirometra* found in 13 species of wild-caught snakes. Letters on the horizontal axis for each column are the abbreviation of snake Latin names: *D.a*—*Deinagkistrodon acutus*, *B.m*—*Bungarus multicinctus*, *N.a*—*Naja atra*, *Di.r*—*Dinodon rufozonatum*, *E.c*—*Elaphe carinata*, *E.t*—*Elaphe taeniura*, *En.b*—*Enhydris bocourti*, *En.c*—*Enhydris chinensis*, *En.p*—*Enhydris plumbea*, *P.k*—*Ptyas korros*, *P.m*—*Ptyas mucosus*, *X.p*—*Xenochrophis piscator*, and *Z.d*—*Zoacys dhumnades*.

Overall, 5,698 worms of* Spirometra* were isolated from 251 snakes, accounting for 55.0% of the total examined snake samples. The exterior view of *Spirometra* was shown in Figure 1. The worm burden per infected snake ranged from 1 to 213, while the prevalence in the 13 examined species was 0~96.2% and the mean infection intensity was 12.5 (Table 2). More than half (58.1%) of the spargana located in muscular tissue, 25.6% in subcutaneous tissue, and 16.3% in the coelomic cavity (Figures 2 and 3). The nonparametric test showed that the density distribution among the muscle tissue, subcutaneous tissue, and coelom was significantly different (*P* < 0.05) (Table 2, Figure 3).

The prevalence and infection intensity of *Spirometra* had large differences among snake species (Figure 3). Snake species in Elapidae had lower prevalence and infection intensity of *Spirometra*. In *Enhydris*, 3 species, *En. bocourti*, *En. chinensis*, and *En. Plumbea*, were even free of *Spirometra* infection. *Spirometra* infection was common prevalence in the snake species, including *Z. dhumnades*, *Di. rufozonatum*, *X. piscator*, *E. carinata*, *P. korros*, *D. acutus*, and *P. mucosus*.

## 4. Discussion

Colubridae and Elapidae are the main target species in food markets in China [10, 13]. In this investigation, we checked 13 common snake species belonging to 3 families (1 species in Viperidae, 2 species in Elapidae, and 10 species in Colubridae). The results indicate that *Spirometra* severely infected wild-caught snakes in food markets in Guangzhou and Shenzhen. More than half (55.0%) of the snakes were infected by *Spirometra* and the mean intensity of infection reached 12.5 worms per snake. Similar results were obtained in the studies of other areas or other species of snakes [12]. Due to the high prevalence of infection in snakes, consuming snakes has great risk of infection by sparganosis.

In this survey, the prevalence and intensity of* Spirometra* infection were different among snake species. There seems to be relevance between *Spirometra* infection and the feeding habits of snakes. Generally, nonpoisonous snakes prefer to prey frogs [11] and these species seem more susceptible to *Spirometra* infection. There is an interesting phenomenon that three species of genus *Enhydris* which dwells in the water and mainly prey on fishes and tadpoles were free of infection by *Spirometra*. In theory, those snakes have more opportunities to contact *Cyclops*, the first intermediate host of *Spirometra*, and are prone to be infected by *Spirometra* through eating the second intermediate hosts (tadpoles and frogs). As a result, we infer these snakes may have some resistance to *Spirometra* and this is worth conducting further study. To further understand the reasons of *Spirometra* infection in snake, more detailed survey on the habitats and dietary habit of these snakes are necessary. However, it is a regret that we cannot trace the source of these snakes. As the centers of snake consumption, snakes in food markets of Guangzhou and Shenzhen may come from not only local but also other provinces, even other countries [14, 15].

In China, there are a lot of cases of human sparganosis caused by eating raw meat of snakes and frogs, drinking snake blood, and swallowing snake gall bladder [16]. Improper cooking methods of snakes will also increase the risk of infection, such as snake skin salad and halfcooked snake meat. In addition, *Spirometra* may contaminate tableware and food in the process of cooking snake meat. In the year of 2011, a patient suffered from bronchial sparganosis because he had a history of ingesting raw frogs, snakes, and drinking raw snake blood [17]. Another case of cerebral sparganosis reported in 2012 was caused by eating frogs and snakes [18]. In a separate report in 2003, all of the 11 patients infected by *Spirometra* had the habit of eating raw meat and skin of animals and 6 of them ate snake meat, blood, or snake gall [19]. In 104 cases from 2000 to 2006, 53.9% were caused by eating snakes or frogs [16].

Our study indicated that eating wild-caught snakes has great health risk and improper cooking methods may increase the risk of *Spirometra* infection. In recent years, eating snakes and other wild-caught animals have resulted in numerous cases of human sparganosis [16]. However, the traditional habit of eating snakes still prevails in southern China, especially in Guangdong Province [8]. Based on this study, we present three suggestions for preventing human sparganosis: (1) the harm and epidemiology of sparganosis should be publicized and popularized, (2) the illegal trade in wild-caught snakes should be effectively controlled and the quarantine of snakes in food markets should be strengthen, and (3) long-term monitoring of the sparganosis of snakes in food markets should be conducted to provide scientific basis for preventing and controlling the sparganosis.

## Figures and Tables

**Table 1:** Essential information of the 456 snake samples from food markets in Guangzhou and Shenzhen, China.

Species of snakes	Source of samples	Number of samples	Range of body length (cm)	Medians of body length (cm)	Range of body weight (g)	Medians of body weight (g)
Viperidae						
* Deinagkistrodon acutus *	Guangzhou	5	105~123	110	665.0~1,155.7	723.8
Elapidae						
* Bungarus multicinctus *	Guangzhou and Shenzhen	13	75~120	100	66.0~295.1	178.2
* Naja atra *	Guangzhou	28	88~173	118.5	167.0~1,925.8	663.95
Colubridae						
* Dinodon rufozonatum *	Shenzhen	44	81~125	98.5	104.5~334.4	162.9
* Elaphe carinata *	Guangzhou	11	146~211	178	831.4~2,167.0	1,121.6
* E. taeniura *	Guangzhou	26	83~163	123	63.7~334.0	178.35
* Enhydris bocourti *	Guangzhou	9	70~85	80	337.5~487.0	406.3
* En. chinensis *	Guangzhou	29	27~72	46	15.3~230.3	53.2
* En. plumbea *	Guangzhou	28	30~47	39	15.1~63.8	27.75
* Ptyas korros *	Guangzhou and Shenzhen	55	91~162	121	109.0~514.0	222.3
* P. mucosus *	Guangzhou and Shenzhen	49	150~240	202	433.3~2,346.0	948
* Xenochrophis piscator *	Guangzhou and Shenzhen	107	53~110	82	26.6~419.9	191.7
* Zoacys dhumnades *	Guangzhou and Shenzhen	52	96~220	172	517.0~1,165.4	606.6

**Table 2:** Prevalence, intensity, and parasitizing locations of *Spirometra* found in 13 species of wild-caught snakes from food markets in Guangzhou and Shenzhen, China.

Species of snakes	Infection of *Spirometra *			Locations of *Spirometra *		
	Prevalence (%)	Intensity of infection	Mean intensity of infection	Muscle	Subcutaneous tissue	Coelom
Viperidae						
* Deinagkistrodon acutus *	60.0	0~34	9.6	32	14	2
Elapidae						
* Bungarusmulticinctus *	7.7	0~5	0.4	0	0	5
* Naja atra *	14.3	0~3	0.3	6	1	1
Colubridae						
* Dinodon rufozonatum *	79.5	0~65	14.4	407	137	89
* Elaphe carinata *	63.6	0~172	42.3	232	180	53
* E. taeniura *	15.4	0~5	0.6	12	0	4
* Enhydris bocourti *	0	0	0	0	0	0
* En. chinensis *	0	0	0	0	0	0
* En. plumbea *	0	0	0	0	0	0
* Ptyas korros *	40.0	0~65	3.3	72	68	43
* P. mucosus *	81.6	0~81	12.0	341	161	88
* Xenochrophis piscator *	79.4	0~133	15.1	1,138	259	216
* Zoacys dhumnades *	96.2	0~213	41.1	1,072	636	429

Total	—	0~213	—	3,312	1,456	930
Means	55.0	—	12.5	7	3	2

Note: Kruskal-Wallis test showed significant difference among the number of worms in muscle, subcutaneous tissue, and coelom (*P* < 0.05).
